# Suicidal ideation in the perinatal period: findings from the Thailand–Myanmar border

**DOI:** 10.1093/pubmed/fdab297

**Published:** 2021-08-03

**Authors:** G Fellmeth, S Nosten, N Khirikoekkong, M M Oo, M E Gilder, E Plugge, M Fazel, R Fitzpatrick, R McGready

**Affiliations:** National Perinatal Epidemiology Unit, Nuffield Department of Population Health, University of Oxford, Oxford, UK; Shoklo Malaria Research Unit, Mahidol-Oxford Tropical Medicine Research Unit, Mae Sot, Thailand; Shoklo Malaria Research Unit, Mahidol-Oxford Tropical Medicine Research Unit, Mae Sot, Thailand; Shoklo Malaria Research Unit, Mahidol-Oxford Tropical Medicine Research Unit, Mae Sot, Thailand; Department of Family Medicine, Faculty of Medicine, Chiang Mai University, Chiang Mai, Thailand; Primary Care, Population Sciences and Medical Education, University of Southampton, Southampton, UK; Department of Psychiatry, University of Oxford, Oxford, UK; Nuffield Department of Population Health, University of Oxford, Oxford, UK; Shoklo Malaria Research Unit, Mahidol-Oxford Tropical Medicine Research Unit, Mae Sot, Thailand; Faculty of Tropical Medicine, Mahidol University, Bangkok, Thailand

**Keywords:** mental health, migration, pregnancy and childbirth disorders

## Abstract

**Background:**

Suicide is a leading cause of maternal death globally. Migrant and refugee populations may experience higher risk of suicide. We report data on suicidal ideation from migrant and refugee women living on the Thailand–Myanmar border.

**Methods:**

Women were recruited in their first trimester of pregnancy. Depression status was assessed by diagnostic interview in the first, second and third trimesters and at 1 month post-partum. We calculated prevalence of suicidal ideation and used logistic regression to identify associated socio-demographic factors.

**Results:**

During the perinatal period, 5.3% (30/568) women experienced suicidal ideation. Refugee women were more likely to experience suicidal ideation than migrant women (8.0 versus 3.1%; *P* = 0.01). Most women with suicidal ideation did not have severe depression. Previous trauma (OR 2.32; 95% CI: 1.70–3.15) and unplanned pregnancy (OR 2.74; 95% CI: 1.10–6.86) were significantly associated with suicidal ideation after controlling for all other variables.

**Conclusions:**

Suicidal ideation represents an important symptom among migrant and refugee women on the Thailand–Myanmar border. Screening only those with severe depression may be insufficient to identify women at risk of suicide. Community-level interventions addressing social and gender inequalities and prioritization of family planning programmes are needed alongside targeted suicide prevention initiatives to help lower the rates of people dying by suicide.

## Introduction

Suicide is a leading cause of maternal death across low- and middle-income countries (LMIC) and high-income countries (HIC) alike.^1–3^ Globally, an estimated 1.7% of pregnancy-related deaths is attributable to suicide, with higher rates of 2.2% reported in Southeast Asia.^1^ Stigma around suicide and mental illness leads to many maternal deaths from suicide being classified as accidental deaths; official figures are therefore unlikely to represent the true extent of the burden.^1^ Marginalized groups including migrant and refugee populations may experience higher risk of suicide as evidenced by high suicide and suicidal ideation rates among migrant populations living in HIC.^4–6^ Among adults who have had suicidal ideation, approximately a quarter will, in their lifetime, make an attempt on ending their lives. Significantly, adults of childbearing age may have a lower threshold for acting on suicidal ideation.^7^^,^^8^ Few studies have examined the risk of maternal suicide in migrant and refugee populations living in LMIC, where access to mental health services is limited.

Decades of civil conflict in Myanmar has resulted in large-scale displacement of populations from Myanmar to the Thai border region. Resettled populations from Myanmar include refugee and migrant populations. Refugee populations live in established refugee camps on the Thai side of the border, having fled poverty and conflict. Many refugees await opportunities for resettlement abroad. Migrant populations live in towns and villages along both sides of the border, typically seeking employment in agriculture, manufacturing and service industries. Many migrant workers in this area lack formal documentation, excluding this group from accessing Thai health and social services and rendering them vulnerable to exploitation and deportation. Among migrant and refugee women on the Thailand–Myanmar border, suicide accounted for 9% of maternal deaths between 1998 and 2015, higher than the general population.^9^^,^^10^ We report data on suicidal ideation from a cohort of migrant and refugee women living in this area between 2015 and 2017.

## Methods

We conducted a prospective cohort study of migrant and refugee women living along the Thailand–Myanmar border.^11^ In brief, women attending antenatal clinics provided by the Shoklo Malaria Research Unit who were aged >18 years, in their first trimester of pregnancy, had a viable pregnancy and were willing and able to take part were invited to participate. Recruitment too place between October 2015 and April 2016. Depression status was assessed in the first, second and third trimesters and at 1 month post-partum using the Structured Clinical Interview for the Diagnosis of DSM-IV Disorders (SCID).^12^ The SCID included one item on suicidal ideation, phrased in the Karen and Burmese SCID as: ‘Do you ever think about suicide?’, with follow-up prompts to elicit details on the nature and frequency of these thoughts, actions taken towards carrying out suicide and previous attempts. SCID criteria categorized suicidal ideation as: absent (no suicidal thoughts reported); subthreshold (occasional abstract or hypothetical thoughts around suicide without active intent) or present (pervasive and severe thoughts around suicide, often with active intent). Sociodemographic variables were collected during pregnancy and after delivery. These included demographic (e.g. age, ethnicity, religion), economic (e.g. employment, income), psychosocial (e.g. previous depression, social support, interpersonal violence, trauma) and migration (e.g. duration since migration) factors. Interviews were conducted by experienced midwives in Karen and Burmese. Interview notes were translated into English and independently reviewed by a clinician. We calculated the prevalence of suicidal ideation and used univariable logistic regression to calculate unadjusted odds ratios of associations between sociodemographic variables and suicidal ideation. Variables associated with suicidal ideation at *P* < 0.05 in univariable analyses were included in a stepwise multivariable logistic regression.

## Results

The cohort included 568 (318 migrant; 250 refugee) women. Detailed characteristics of participants have been published previously.^11^ In brief, the median age of participants was 25 years, the predominant ethnicity was Burman among migrant women (50%; 159/318) and Sgaw Karen among refugee women (70.4%; 176/250), and almost half of all participants (45.4%) had completed fewer than 3 years of education. During the perinatal period, 5.3% (30/568) of all participants experienced suicidal ideation and 23.8% (135/568) had subthreshold symptoms (such as occasionally thinking about taking their own life but with no plans to do so). Refugee women were more likely to experience suicidal ideation than migrant women (8.0 versus 3.1%; *P* = 0.01). Women with suicidal ideation were 24 times more likely than women without suicidal ideation to have a diagnosis of depression (OR 24.04; 95% CI: 5.67–102.00; *P* < 0.01). Of the 30 women with suicidal ideation, only 43.3% (13/30) had severe depression and the remaining had either no (*n* = 2), mild (*n* = 2) or moderate (*n* = 13) depression. Table 1 lists variables which were statistically significantly associated with suicidal ideation in the univariable analysis (non-significant variables are provided in the table footnote). In the multivariable model, only trauma score (adjusted OR 2.32; 95% CI: 1.70–3.15 for every unit increase in score; *P* = 0.03) and unplanned pregnancy (adjusted OR 2.74; 95% CI: 1.10–6.86; *P* < 0.01) remained significantly associated with suicidal ideation.

**Table 1:** Associations between sociodemographic and suicidal ideation (variables significantly associated in univariable and multivariable logistic regression; n=568)

	**Univariable logistic regression** ^a^		**Multivariable logistic regression** ^b^	
	OR (95% CI)	p-value	OR (95% CI)	p-value
Migrant Refugee	1.00 2.68 (1.23–5.83)	0.01	–	–
Household size ^ⱡ^	1.17 (1.01–1.35)	0.03	–	–
Pregnancy planned Pregnancy unplanned	1.00 4.18 (1.80–9.71)	<0.01	1.00 2.74 (1.10–6.86)	<0.01
**Future plans** Return to Myanmar Remain in Thailand Resettle abroad	1.00 3.57 (1.02–12.49) 5.51 (1.77–17.12)	0.05 0.04	–	–
Interpersonal violence^c^	3.39 (1.07–10.68)	0.04	–	–
History of depression^d^	2.29 (1.09–4.85)	0.03	–	–
Trauma score ^ⱡ^^e^	2.28 (1.72–3.02)	<0.01	2.32 (1.70–3.15)	0.03
Social support ^ⱡ^^f^	0.83 (0.72 (0.95)	<0.01	–	–

^a^Other variables entered into univariable logistic regression were not statistically significant: ethnicity, religion, marital status, education, literacy, language spoken, employment, telephone ownership, identification status, country of birth, country of residence, duration in current location, reason for migration, alcohol, smoking, use of betel, parity.

^b^denotes variables dropped from multivariable regression model due to non-significance

^c^Assessed using single question: “Does a partner or anyone at home hurt, hit or threaten you?”

^d^Self-report

^e^Assessed using modified Trauma History Screen^21^; scores 0 (no trauma) to 9 (maximum trauma)

^f^Assessed using modified *Social Support in the Postpartum Period Scale*^22^; scores 0 (poor support) to 8 (high support)

Continuous variables

## Discussion

### Main findings of this study

Suicidal ideation represents an important symptom among migrant and refugee women on the Thailand–Myanmar border. Our results suggest 5% of women in this setting experienced suicidal ideation. Additionally, almost one in four women experienced subthreshold suicidal symptoms. Suicidal ideation was higher among refugee women (8%) than migrant women (3%). While reasons for this difference need further research, it is possible that refugee women experience a greater sense of hopelessness with regards to their futures as a result of limited prospects for resettlement. In contrast, migrant women may feel a stronger sense of control over their futures.^13^ In a previous analysis of data from this cohort, we found that migrant women were more likely to suffer from depression than refugee women: in light of this, the higher level of suicidal ideation among refugee women is surprising.^14^ Further research is needed in this area, including a qualitative exploration of the cultural manifestations of distress. Suicidal ideation was significantly associated with unplanned pregnancy and trauma history. Over half of women with suicidal ideation had either no depression or mild or moderate depression as diagnosed using a clinical interview.

### What is already known on this topic

Our prevalence is similar to that reported in a study of (non-perinatal) Karenni refugee women in northern Thailand, which found rates of suicidality of 7.4%.^15^ A global review of suicidality among perinatal women found rates ranging from 4% in Finland to 15% in India.^16^ The distribution of suicidal ideation across the spectrum of depression severity has important clinical implications and suggests that screening only those with severe depression may be insufficient to identify women at risk of suicide. These findings have been replicated elsewhere. For example, a study of perinatal women in the USA reported that over half of those with suicidal ideation had screened negative for depression, while a study of Burmese refugees in Thailand found that suicidal thoughts were a poor indicator of depression.^8^^,^^17^ In other settings, similar associations between suicidal ideation and psychosocial factors have been highlighted. Among pregnant women in India, for example, suicide was associated with low levels of social support, domestic violence, depressive symptoms and previous suicidality.^18^ Alongside depression screening, a broader psychosocial assessment may help to identify women at risk of suicide.

### What this study adds

High rates of suicidal ideation, the significant number of maternal suicides and the severe consequences of these tragic events on families, children and communities make suicide prevention an urgent priority on the Thailand–Myanmar border. Evidence around suicide prevention among displaced populations remains limited.^19^ Our findings suggest that initiatives to address previous trauma experiences as well as increased investment in and prioritization of family planning programmes for migrant and refugee women are needed to address suicide in this region. Our results serve as a reminder that from a population perspective, most suicides occur in lower risk populations and prevention efforts should not focus solely on high-risk individuals.^8^ More broadly, there is a need to wider community-level interventions. Accurate data collection and monitoring systems as well as initiatives to destigmatize mental illness and encourage help-seeking behaviour are important, alongside building connectivity and social networks, forging opportunities for individuals to contribute to the social and economic capital of a community, investment in schools and good quality childcare and promoting social and gender equality—all of which are likely to contribute to lowering the rates of people dying by suicide.^20^ In order for such initiatives to be sustainable and effective, political resolve on a national level as well as resource investment at the community level are essential.

### Limitations of this study

Although the SCID is a robust diagnostic tool, stigma around mental disorders may have led to under-reporting of suicidal thoughts and thus an under-estimation of the true extent of the burden. A further limitation is that because women with depression were offered treatment, SCID responses after the baseline assessment are likely to under-estimate the true level of suicidal ideation in this population.

## Data Availability

The data underlying this article will be shared on reasonable request to the corresponding author.

## Notes

**G. Fellmeth**, Dr

**S. Nosten**, Ms

**N. Khirikoekkong**, Ms

**M.M. Oo**, Ms

**M.E. Gilder**, Dr

**E. Plugge**, Dr

**M. Fazel**, Associate Professor

**R. Fitzpatrick**, Professor

**R. McGready**, Professor
